# Soil Actinobacteria Exhibit Metabolic Capabilities for Degrading the Toxic and Persistent Herbicide Metribuzin

**DOI:** 10.3390/toxics12100709

**Published:** 2024-09-29

**Authors:** Hadjer Rebai, Essam Nageh Sholkamy, Mohamed A. A. Abdelhamid, Pratheesh Prakasam Thanka, Ashraf Aly Hassan, Seung Pil Pack, Mi-Ran Ki, Allaoueddine Boudemagh

**Affiliations:** 1 Laboratory of Molecular and Cellular Biology, Constantine 1—Frères Mentouri University, Chaâbat Erssas Campus, Ain El Bey Road, Constantine 25000, Algeria; hadjer.rebai@umc.edu.dz (H.R.);; 2 Department of Microbiology, Constantine 1—Frères Mentouri University, Chaâbat Erssas Campus, Ain El Bey Road, Constantine 25000, Algeria; 3 Department of Botany and Microbiology, College of Science, King Saud University, P.O. Box 2455, Riyadh 11451, Saudi Arabia; 4 Biology Department, Faculty of Education and Arts, Sohar University, Sohar 311, Oman; mohamed42@korea.ac.kr; 5 Department of Biotechnology and Bioinformatics, Korea University, Sejong-ro 2511, Sejong 30019, Republic of Korea; spack@korea.ac.kr (S.P.P.); allheart@korea.ac.kr (M.-R.K.); 6 Department of Civil and Environmental Engineering, United Arab Emirates University, Al Ain P.O. Box 15551, United Arab Emirates; pratheesh.p@uaeu.ac.ae (P.P.T.); alyhassan@uaeu.ac.ae (A.A.H.); 7 Institute of Industrial Technology, Korea University, Sejong-ro 2511, Sejong 30019, Republic of Korea

**Keywords:** triazine, metribuzin, biodegradation, microorganisms, *Streptomyces*, agricultural soil

## Abstract

Metribuzin, a widely used triazine herbicide, persists in agricultural soils and poses significant environmental pollution threats globally. The aim of this study was to investigate the biodegradation of metribuzin by actinobacterial strains in vitro at different environmental conditions. From an initial screen of 12 actinobacterial strains, four bacteria exhibited robust growth in the presence of the metribuzin as the sole carbon source at 50 mg/L concentration. The optimization of metribuzin biodegradation under different conditions (pH, temperature and inoculum size) using a spectrophotometric method revealed that maximum degradation of metribuzin occurred at a pH of 7.2, a temperature 30 °C, and at an inoculum volume of 4%. Subsequent GC-MS validation confirmed the remarkable biodegradation capabilities of the actinobacterial isolates, where the strain C1 showed the highest rate of metribuzin degradation of 83.12%. Detailed phylogenetic identified the active strains as *Streptomyces toxytricini* (CH), *Streptomyces stelliscabiei* (B2), and two *Streptomyces heliomycini* (C1, C3). Structural analysis by ATR-FTIR spectroscopy confirmed the extensive biotransformation of the herbicide molecule. Our findings highlight the immense untapped potential of soil actinobacteria, particularly the *Streptomyces heliomycini* C1 strain, as versatile bioremediation agents for removing persistent agrochemical pollutants.

## 1. Introduction

Metribuzin (MB), a widely used triazine herbicide, has become a pervasive environmental contaminant due to its high water solubility, limited soil adsorption, and recalcitrance to degradation. With a chemical structure of 4-amino-6-tert-4, 5-dihydro-3-methylthio-1, 2, 4-triazin-5-one, this photosystem II inhibitor effectively controls grassy weeds and broadleaf species in major crop systems like tomato, corn, sugarcane, and potato [1,2,3,4,5,6]. Triazine herbicides, including metribuzin, are widely applied in agriculture to increase crop yields and protect against weed infestations. However, their high mobility and persistence pose significant risks of surface and groundwater contamination, as well as toxicity to non-target organisms [7,8,9,10,11]. These environmental concerns have prompted a growing need to develop effective remediation strategies to mitigate the impact of metribuzin pollution.

Bioremediation, which leverages the metabolic capabilities of microorganisms, represents a promising approach to addressing these environmental impacts [12]. Microorganisms possess diverse catabolic pathways that allow them to utilize a wide range of organic compounds, including pesticides and other xenobiotics, as carbon and energy sources.

Microorganisms degrade metribuzin, resulting in the production of various metabolites, such as desamino-metribuzin (DA), diketometribuzin (DK), and desamino-diketometribuzin (DADK) [13]. Research conducted by Mutua et al. [14], Khoury et al. [15] and Henriksen et al. [8], indicates that metribuzin can undergo deamination and desulfuration, leading to the formation of DA and DK, respectively. Additionally, the deamination of DK and DA desulfuration yield DADK. While the specific genes responsible for metribuzin biodegradation have not been extensively studied, several genes associated with the breakdown of s-triazines—such as *atzA*, *atzB*, *atzC*, *atzD*, *atzE*, and *atzF*—are well documented [16,17].

While various bacteria, fungi, and algae have demonstrated the ability to degrade triazine herbicides [18,19,20,21], research on metribuzin-degrading microbes remains limited [6,22,23,24,25,26,27,28]. Identifying and characterizing microbes capable of efficiently degrading metribuzin is crucial for developing effective bioremediation solutions.

Actinobacteria, as versatile and metabolically adept Gram-positive bacteria, have garnered significant interest as promising bioremediation agents [28]. These bacteria are known for their diverse catabolic capabilities, enabling them to degrade a wide range of recalcitrant organic compounds, including pesticides and xenobiotics. Actinobacteria are commonly found in soil environments and are recognized for their ability to adapt to various stress conditions, making them well suited for bioremediation applications. However, few studies have explored the potential of actinobacteria to degrade metribuzin specifically [13,23,25]. The agricultural soils of El-Oued, a Saharan region with extensive metribuzin use, may harbor adapted actinobacterial populations capable of efficiently metabolizing this persistent herbicide.

In this study, we sought to investigate the potential of actinobacterial strains isolated from El-Oued soils to degrade metribuzin. To study the optimization of the critical environmental parameters governing this bioremediation process, a kinetic analysis was conducted. This research has the potential to contribute to the development of sustainable and environmentally friendly solutions for managing the impact of metribuzin pollution on ecosystems and human health.

## 2. Materials and Methods

### 2.1. Sample Collection and Actinobacteria Isolation

The El-Oued (Guemar) site was selected for this study due to its significance in Algerian agriculture, characterized by a variety of crops and high levels of pesticide usage. Located in the northeastern region of the Algerian Sahara, near the Algerian-Tunisian border, Guemar is a commune within the El Oued wilaya, situated at coordinates 33°29′32″ N and 6°47′50″ E.

A soil sample was collected from a potato field in El-Oued, Algeria, following the method outlined by Aouar et al. [29]. Specifically, 100 to 150 g of soil was extracted from a depth of 5 to 15 cm. After sieving to remove debris such as stones and roots, approximately 50 g of the soil was transported to the laboratory, maintaining a temperature of 4 °C. Actinobacteria were isolated from the sample using the serial dilution technique. One gram of soil was diluted in 9 mL of physiological saline, and 0.1 mL aliquots of the dilutions were spread onto yeast –malt extract–Agar [30] and Bennett media [31], supplemented with 75 μg/mL amphotericin B to inhibit fungal growth. The inoculated plates were incubated at 30 °C for 21 days, and the resulting actinobacterial colonies were selected based on morphological characteristics, purified on antifungal-free Bennett medium, and stored at −4 °C [32,33].

### 2.2. Screening for Metribuzin-Degrading Actinobacteria

The purified actinobacterial isolates were evaluated for their ability to utilize the herbicide metribuzin as a sole carbon source. Mineral salt medium (MSM) (Table S1) agar plates were supplemented with metribuzin at concentrations ranging from 1 to 200 mg/L, and the isolates were surface-inoculated and incubated at 30 °C for one week. Positive growth indicated the capacity to degrade metribuzin, and the isolates that grew at the highest herbicide concentration were selected for further testing in a liquid MSM medium containing 50 mg/L metribuzin [34,35].

### 2.3. Actinobacterial Identification

#### 2.3.1. Morphological, Biochemical, and Physiological Characterization

The selected actinobacterial isolates were characterized according to standard protocols. Colony morphology was determined on ISP2 medium (International *Streptomyces* Project 2) [36] after 15 days of incubation at 30 °C. The growth and macroscopic characteristics were determined on ISP2, ISP7, ISP9, Bennett and yeast malt extract Agar media (detailed information on the medium is reported in Table S1), Carbon source utilization [36], nitrogen source assimilation [37], and other biochemical tests were performed using established methods by Tatsinkou Fossi et al. [38], Li et al. [39], and Minotto et al. [40]. Tolerance to various pH levels, temperatures, and NaCl concentrations was assessed using ISP2 medium.

#### 2.3.2. Molecular Identification

For molecular identification, actinobacterial isolates were cultured in ISP2 medium for four days. DNA extraction was performed using the DNeasy Blood and Tissue Kit (250) (BioRad T100, Foster City, CA, USA), following the manufacturer’s instructions. PCR amplification of the 16S rRNA gene was conducted in an automated thermal cycler (BIO-RAD T100) with a universal primer set, 27F: 50 AGAGTTTGATCMTGGCTCAG-30 and 1492R:50-TACGGYTACCTTGTTACGACTT-30.

The amplification reaction was prepared in a total volume of 20 µL using the 5× HOT FIREPol^®^ Blend Master Mix. The thermal cycling program included an initial denaturation at 95 °C for 5 min, followed by 35 cycles of denaturation at 95 °C for 30 s, annealing at 50 °C for 40 s, and extension at 72 °C for 2 min, concluding with a final extension at 72 °C for 7 min, with slight modifications [41]. The resulting 16S rRNA gene sequences were deposited in GenBank for comparison with existing sequences.

Phylogenetic analysis was conducted using MEGA11 software (version 11.0.13) and the neighbor-joining method.

### 2.4. Metribuzin Biodegradation Kinetics and Optimization

The biodegradation of metribuzin by the selected actinobacterial isolates was evaluated in a liquid MSM medium containing 50 mg/L of the herbicide. Bacterial growth was determined by measuring the dry weight, and the residual metribuzin concentration was quantified using a UV–visible spectrophotometer [34]. The effects of pH (4, 6, 7.2, 9) (adjusted by HCl (2M) and NaOH (5M), temperature by incubator setting (10 °C, 25 °C, 30 °C, 37 °C), and inoculum size (2%, 4%, 7%, 9% *w*/*v*) on the biodegradation process were investigated [3].

### 2.5. Chemical Analysis

#### 2.5.1. Metribuzin Residue Detection

Residual metribuzin concentrations in the samples were determined using GC-MS analysis. The samples were centrifuged, filtered, and injected into the GC-MS system equipped with an HP-5ms ultra-inert capillary column and operated under optimized conditions [42].

#### 2.5.2. Total Organic Carbon Analysis

The amount of organic carbon reduced after metribuzin biodegradation was measured using a TOC-L Shimadzu analyzer (Shimadzu, Kyoto, Japan). After 15 days of incubation, the samples were centrifuged, filtered, and diluted before analysis [43].

#### 2.5.3. ATR-FTIR Analysis of Metribuzin

The degradation of the herbicide metribuzin was monitored using state-of-the-art ATR-FTIR spectroscopy (Thermo Fisher Scientific Inc., Madison, WI, USA). Metribuzin samples were analyzed on a high-performance ATR-FTIR instrument following a 15-day incubation period. Briefly, 1 mL aliquots were collected and centrifuged at 10,000 rpm and 4 °C for 15 min, and the resulting supernatants were filtered through 0.45 μm membranes. The filtered supernatants were then directly analyzed without further sample preparation. A drop of each supernatant was carefully placed on the ATR crystal window, ensuring intimate contact with the crystal surface under light pressure. Spectral data were acquired in the mid-infrared range from 500 to 4000 cm^−1^. The obtained spectra were compared to reference data to identify the presence of metribuzin and potential degradation products [44,45].

### 2.6. Kinetic Analysis

The kinetic parameters governing the biodegradation of metribuzin were determined by plotting the natural logarithm of the relative concentration (ln [C_t_/C_0_]) against time (days). The degradation rate constant (k) and half-life (T_1/2_) were calculated using the following well-established equations:

(1) $$C_{t}=C_{0}\;\times \;e-\mathrm{kt}$$

(2) $$T_{1/2}\;=\;\ln2/k$$

C_0_ means the concentration of metribuzin at t_0_, C_t_ means the concentration of metribuzin at time t, and k is the degradation rate constant (day^−1^).

### 2.7. Statistical Analysis

All experiments were performed in triplicate, and the results are presented with a standard error. Two-way ANOVA and Tukey’s post hoc tests were employed to determine statistical significance, with a threshold of *p* ≤ 0.05.

## 3. Results

### 3.1. Isolation and Preliminary Screening of Metribuzin-Degrading Actinobacteria

Twelve actinobacterial strains were successfully isolated from metribuzin-contaminated soils (Table 1). Remarkably, four isolates (CH, B2, C1, and C3) exhibited robust growth on a minimal solid medium supplemented with 50 mg/L of metribuzin, indicating their remarkable ability to utilize this herbicide as a sole carbon source.

### 3.2. Phenotypic Characterization of the Active Isolates

Macroscopic examination of the cultures revealed that all isolates displayed morphological features characteristic of the actinobacteria (Table S2), including the presence of both substrate and aerial mycelia. Microscopic analysis confirmed that the isolates were Gram-positive and filamentous. The bacteria thrived on various media, including ISP2, Bennett, and yeast malt extract Agar, but showed poor growth on ISP7 and ISP9. Interestingly, three isolates (B2, C1, C3) produced a dark-brown diffusible pigment, while strain CH did not. Strain CH formed a distinctive pink aerial mycelium with a light pink substrate, whereas the other three isolates exhibited dark-gray aerial and brown substrate mycelia. Carbohydrate utilization profiles revealed that all isolates could metabolize D-glucose and D-galactose, but the CH strain could not utilize D-fructose. Remarkably, not all the nitrogen sources were utilized by the actinobacterial isolates.

The isolates exhibited exceptional adaptability to various environmental conditions. They grew abundantly at sodium chloride concentrations 2%. However, at the concentration of 5%, only strain CH grew. Furthermore, the bacteria displayed remarkable pH tolerance, thriving across the entire pH range tested, with optimal growth at neutral pH. Temperature profiling showed that the isolates grew optimally at 30 °C, with abundant growth from 25 °C to 37 °C, but minimal growth at 40 °C (Table 2).

### 3.3. Molecular Characterization of Metribuzin-Degrading Actinobacterial Isolates

**Figure 1:**
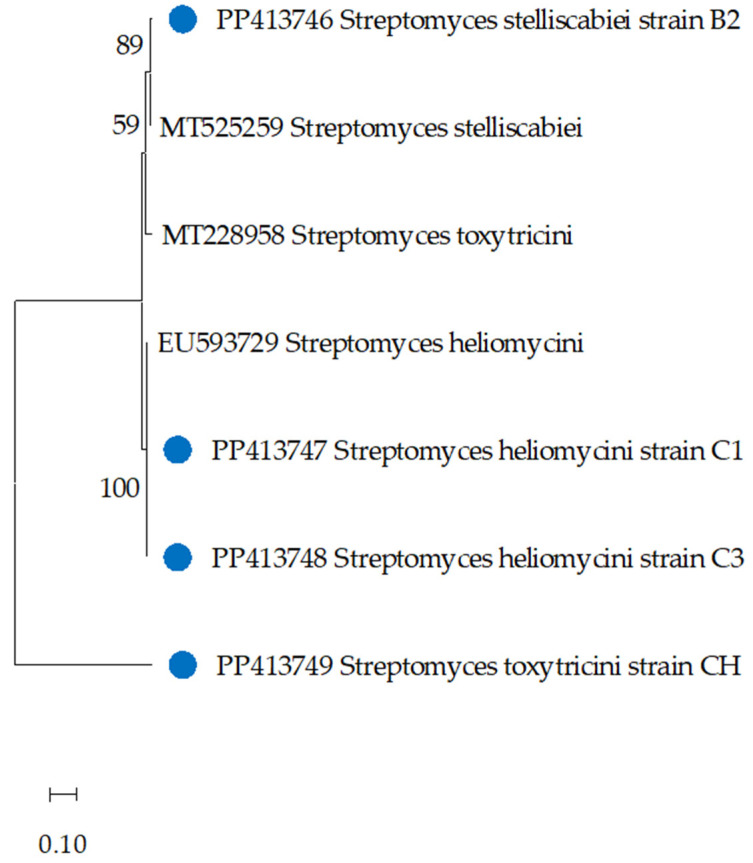
Neighbor-joining phylogenetic tree based on 16S rRNA gene sequences, showing the nearest neighbors of the isolated strains. GenBank accession numbers are indicated next to the branches. Blue dot labels bacterial species.

The 16S rRNA gene sequencing analysis provided precise taxonomic identification of the actinobacterial isolates capable of degrading the herbicide metribuzin (Table 3). The phylogenetic analysis revealed that isolates CH, B2, C1, and C3 belong to the genus *Streptomyces*, a prolific group of soil-dwelling actinobacteria known for their diverse metabolic capabilities. Interestingly, the phylogenetic tree (Figure 1) showed that strain CH formed a distinct clade, suggesting it may represent a novel *Streptomyces* species. Further genetic studies will be required to confirm the taxonomic novelty of this exceptional metribuzin-degrading isolate. Strain B2 was closely related to *Streptomyces stelliscabiei*, with a 90% sequence similarity, indicating a close evolutionary relationship to this known *Streptomyces* species. In contrast, the phylogenetic analysis placed strains C1 and C3 in a distinct clade, with a 100 and 93% sequence similarity to the type of strain of *Streptomyces heliomycini*, respectively.

### 3.4. Growth of Isolates in the Presence of Metribuzin

**Figure 2:**
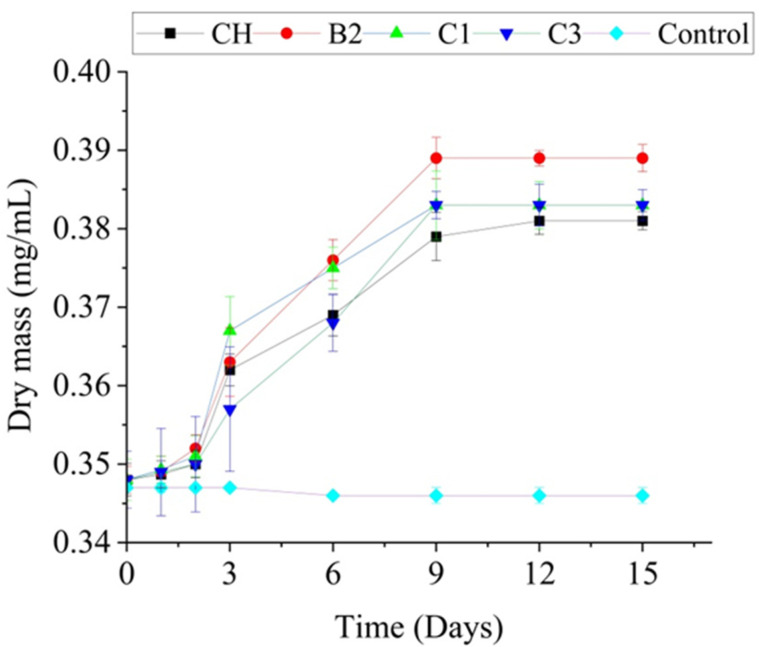
Growth kinetics of the strains CH, B2, C1, and C3 with metribuzin at the concentration of 50 mg/L.

Screening on solid media revealed that all four isolates (CH, B2, C3, C1) exhibited good growth at metribuzin concentrations ranging from 1 to 50 mg/L as the sole source of carbon. Furthermore, kinetic studies in liquid medium supplemented with 50 mg/L metribuzin demonstrated that the isolates were able to rapidly utilize the herbicide as the sole carbon source, with a lag phase in the first 2 days of incubation. Among the active strains, B2 exhibited the most robust growth, with a 4.1% increase, followed by C1 and C3 with a 3.5% increase, and CH with a 3.3% increase (Figure 2). No significant difference was observed in the growth of actinobacterial isolates CH, B2, C1 and C3 in the presence of metribuzin at 50 mg/L (*p*-value > 0.05).

### 3.5. Biodegradation Kinetics and Optimization Insights

**Figure 3:**
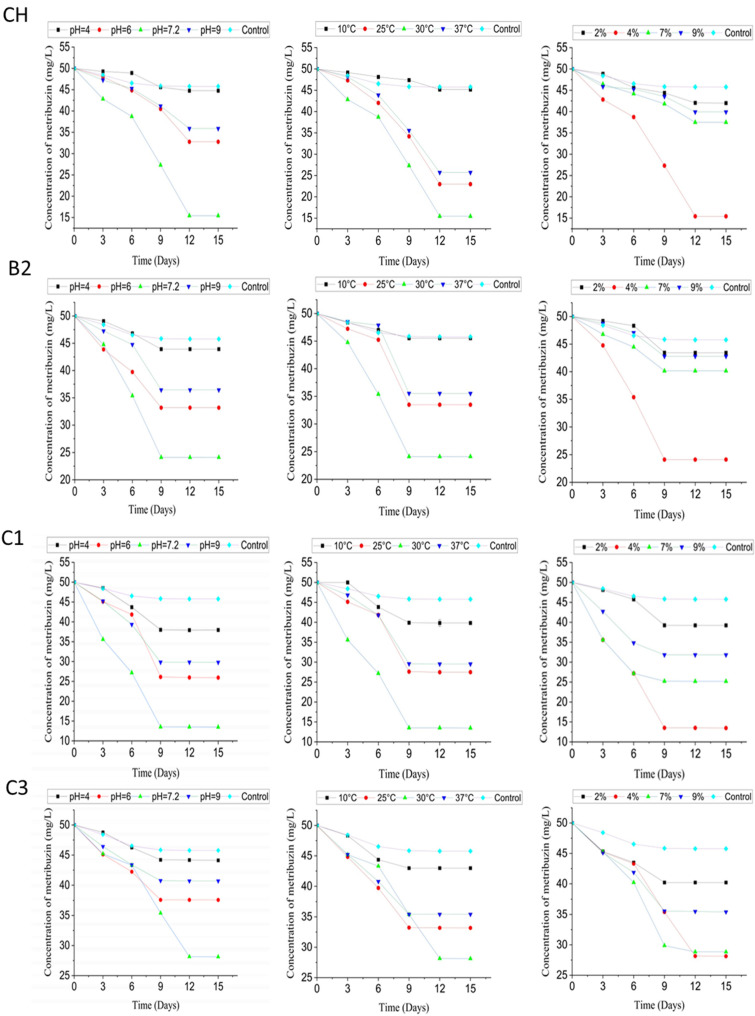
Determination of the degraded metribuzin amount by the strains CH, B2, C1 and C3 after 15 days of incubation, at different pH, different temperatures, and different inoculum volumes, with the abiotic control.

The results of metribuzin biodegradation by bacteria CH, B2, C1, and C3 under different pH conditions, temperatures, and inoculum volumes are presented in Figure 3.

The data reveal that neutral pH (7.2) serves as the optimal pH, with CH, B2, C1, and C3 exhibiting peak degradation rates of 69.14%, 51.74%, 73.06%, and 43.74%, respectively. Interestingly, the C1 strain emerged as the frontrunner, achieving the highest biodegradation rate of 73.06%.

Temperature optimization revealed that 30 °C represents the sweet spot for maximal metribuzin elimination, with the aforementioned isolates displaying degradation percentages ranging from 43.74% to 73.06%. The versatility of these strains is further underscored by their ability to maintain respectable degradation efficiencies at lower temperatures, showcasing their potential for diverse environmental applications.

Delving deeper into the optimization process, we observed that a 4% bacterial inoculum proved to be the most effective, unlocking the highest biodegradation rates across all four isolates. Intriguingly, deviations from this optimal inoculum volume resulted in a notable decline in the degradation potential. The parameters pH, temperature, and inoculum volume had a significant impact on the rate of metribuzin degradation by the CH, B2, C1, and C3 bacteria throughout all days of incubation (*p*-value < 0.0001).

### 3.6. Analysis of Metribuzin Biodegradation by GC-MS

Our GC-MS analysis confirmed the remarkable metribuzin elimination capabilities of these bacterial workhorses. After 15 days of incubation under optimal conditions, CH, B2, C1, and C3 were able to degrade an impressive 80.38%, 76.88%, 83.12%, and 79.52% of the initial 50 mg/L metribuzin concentration, respectively.

Detailed kinetic modeling revealed that the degradation process follows second-order kinetics, underscoring the robustness and predictability of this bioprocess. Notably, the C1 strain emerged as the frontrunner, exhibiting the highest rate constant and the lowest half-life, outperforming the other isolates (Table 4).

### 3.7. Evaluation of Total Organic Carbon (TOC)

Further insights were gained through the evaluation of total organic carbon (TOC) reduction, which showcased the efficient mineralization of metribuzin by these bacterial workhorses. After 15 days, the TOC reduction reached as high as 75.99% for the C1 strain, highlighting the comprehensive degradation capabilities of these isolates as in Figure S1.

### 3.8. ATR-FTIR Analysis of Metribuzin Biodegradation

**Figure 4:**
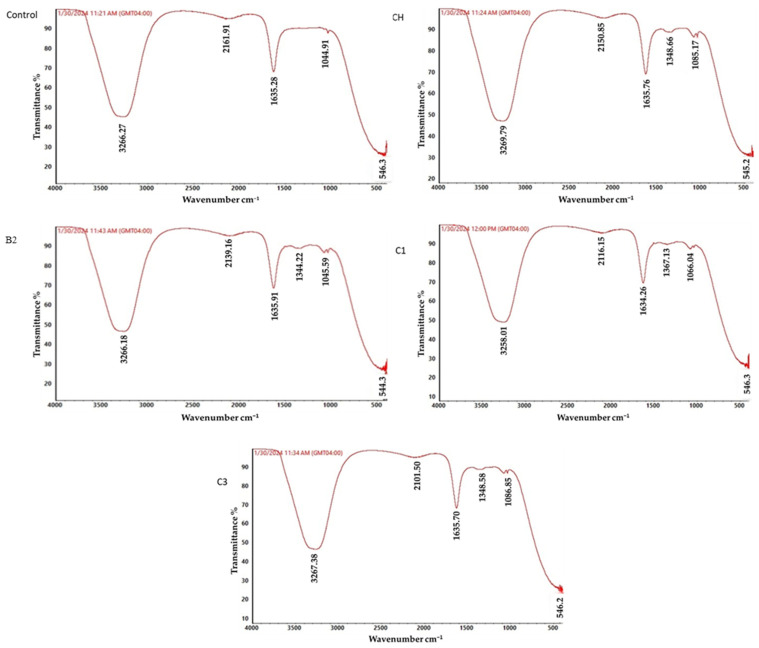
ATR-FTIR analysis of metribuzin at the control (untreated with actinobacterial strains) and after biodegradation by the actinobacterial strains CH, B2, C1, C3.

The structural changes in metribuzin and its biodegraded metabolites produced by various bacterial strains are shown in Figure 4. The characteristic C-O bond of metribuzin was observed at 1044.911 cm^−1^ in the control sample, and was also detected in the spectra of bacteria CH, B2, and C3 at the wavenumber 1045 cm^−1^. Interestingly, this peak was absent in the spectrum of bacteria C1, indicating a potential alteration in or cleavage of the C-O bond during the biodegradation process. Furthermore, the appearance of new peaks from 1085 to 1086 cm^−1^ in the bacterial samples CH, B2, C1, and C3 suggests the formation of C-N bonds, which were not present in the metribuzin control. This observation implies the occurrence of structural modifications, likely involving the introduction of nitrogen-containing functional groups, during the bacterial transformation of metribuzin. Notably, additional peaks were observed at wavenumbers from 1344 cm^−1^ to 1357 cm^−1^ in the spectra of bacteria CH, B2, C1, and C3. These peaks, which were absent in the metribuzin control, may correspond to C-H or O-H vibrations, suggesting the formation of new functional groups or an alteration in the existing ones during the biodegradation process. The presence of peaks at 1634 cm^−1^ and 1635 cm^−1^ in the control and all bacterial samples indicates the persistence of the NH group, a characteristic feature of the metribuzin molecule, throughout the biodegradation reactions. Interestingly, the bacterial strains CH, B2, C1, and C3 exhibited peaks corresponding to the C-O bond at wavenumbers from 2101 cm^−1^ to 2150.853 cm^−1^, which differed from the C-O peak observed at 2160 cm^−1^ in the metribuzin control. These shifts in the C-O bond frequency suggest structural modifications in or the formation of new C-O moieties during the biodegradation process. Finally, the O-H bond, as indicated by the peak at 3266 cm^−1^ in the metribuzin control, was detected in the bacterial samples CH, B2, C1, and C3 at wavenumbers from 3258 cm^−1^ to 3269 cm^−1^, with subtle differences in peak intensity. Overall, the ATR-FTIR analysis revealed significant structural changes in metribuzin upon biodegradation by the various bacterial strains.

## 4. Discussion

The intensive use of herbicides in modern agriculture has become a growing global concern in recent decades [46]. Regions such as El-Oued in Algeria, characterized by their arid climate, have established highly productive agricultural systems that heavily depend on pesticide application, including the herbicide metribuzin. However, the extensive and continuous use of this herbicide has resulted in significant environmental contamination. Recent research has revealed that commercial formulations of metribuzin often contain additives, including adjuvants and metals, which further contribute to soil pollution. The accumulation of these substances poses risks to non-target organisms. To address these challenges, it is essential to implement various bioremediation strategies [47,48].

Various microorganisms, including bacteria such as *Burkholderia* sp., *Pseudomonas* sp., *Bacillus* sp., and fungi like *Pycnoporus coccineus*, have been explored for their metribuzin-degrading capabilities [18,49,50]. However, the scientific literature on the potential of actinobacteria in this regard remains sparse. Notably, Sharma and Saroop [23] have reported the use of a few *Streptomyces aureofaciens* strains for metribuzin biodegradation.

In the present study, we isolated twelve actinobacteria from agricultural soils in El-Oued and identified four isolates capable of utilizing metribuzin as the sole carbon source in a minimal salt medium (MSM) at a concentration of 50 mg/L. These four isolates were further characterized using a polyphasic approach and assigned to the following *Streptomyces* species: *Streptomyces toxytricini* strain CH, *Streptomyces stelliscabiei* strain B2, *Streptomyces heliomycini* strain C1, and *Streptomyces heliomycini* strain C3. When cultured in liquid MSM medium, the selected actinobacteria exhibited limited growth, which may be attributed to the absence of a readily available carbon source, such as glucose, commonly used in similar studies. The lack of a supplementary substrate imposed a metabolic challenge, requiring the bacteria to adapt to metribuzin as the sole carbon source, a process that likely necessitated an extended acclimation period.

To optimize the biodegradation of metribuzin by these actinobacterial isolates, we investigated the influence of various environmental and cultivation parameters, including pH, temperature, and inoculum size, using a simple spectrophotometric method for comparison with the chromatographic analysis under optimal conditions. The results demonstrated that the strains CH, B2, C1, and C3 exhibited the highest biodegradation rates at a neutral pH of 7.2, a temperature of 30 °C, and an inoculum size of 4%. Our results highlight the exceptional adaptability of these bacterial strains, illustrating their capacity to effectively degrade metribuzin across a range of conditions.

Specifically, *Streptomyces toxytricini* strain CH, *Streptomyces stelliscabiei* strain B2, *Streptomyces heliomycini* strain C1, and *Streptomyces heliomycini* strain C3 achieved biodegradation rates of approximately 69.14%, 51.74%, 73.06%, and 43.74%, respectively, under these optimal conditions. The abiotic control resulted in the degradation of 8.44% of the initial concentration of 50 mg/L metribuzin, which can be attributed to factors related to the incubation environment, including light exposure. These findings suggest that the selected actinobacteria are neutrophilic and well-adapted to a neutral environment, consistent with previous studies reporting high metribuzin degradation at neutral pH levels [3,6].

The observed temperature optimum of 30 °C indicates that the isolates are mesophilic, in agreement with the findings of Zhang et al. [6], who reported the highest metribuzin degradation at this temperature. Furthermore, the inoculum size of 4% was found to be optimal for the biodegradation of metribuzin by the actinobacterial strains, corroborating the observations of Zhang et al. [6] that both higher and lower inoculum volumes can negatively impact the degradation efficiency.

The superior metribuzin biodegradation performance of the selected actinobacterial strains under the optimized conditions was further confirmed by GC-MS analysis, which revealed degradation rates ranging from 76.88% to 83.12% of the initial 50 mg/L metribuzin concentration over a 15-day period. Our comprehensive analyses, employing both spectrophotometric and advanced GC-MS techniques, have provided powerful validation of the exceptional metribuzin biodegradation capabilities exhibited by the tested actinobacterial isolates. While the spectrophotometric assessments under optimized conditions revealed remarkable degradation ranging from 43% to 73%, the GC-MS-based validation further elevated these findings, demonstrating biodegradation efficiencies between 76.88% and 83.12% for the same strains. The observed discrepancy between the spectrophotometric and GC-MS results can be attributed to the superior precision and accuracy of the GC-MS analytical method. Previous studies have consistently demonstrated the heightened sensitivity and reliability of GC-MS in quantifying trace-level organic pollutants, including herbicides, compared to conventional spectrophotometric approaches [51]. By leveraging the advanced capabilities of GC-MS, we have been able to more rigorously validate the biodegradation potential of the actinobacterial isolates, underscoring the true magnitude of their remarkable performance.

The current findings demonstrate the exceptional biodegradation capabilities of the isolated actinobacteria strains, which surpass the results reported in previous studies on metribuzin degradation. In contrast to the modest degradation rates observed with other bacterial species, such as the degradation of 14.8% metribuzin by *Bacillus* sp. A6 over 4 days [52] and the 86% metribuzin degradation by *Burkholderia cepacia* strain CH9 over 20 days [22], the actinobacteria isolates in this study achieved remarkably high degradation levels. The only comparable work reported to date is that of Huang et al. [53], who observed 86.4% degradation of 50 mg/L metribuzin by the Gram-negative bacterium *Paracoccus* sp. QCT6 within 7 days. The exceptional biodegradation performance was confirmed by two complementary analytical techniques. Firstly, the total organic carbon (TOC) analysis revealed significant reductions in organic carbon content, reaching up to 75.99% for the C1 isolate. This substantial decrease in organic carbon indicates the occurrence of complete mineralization of the pesticide by the *Bacillus aryabhattai*, *Pseudomonas azotoformans* and *Sphingomonas pseudosanguinis* strains, a finding that aligns with previous reports on the biodegradation of other herbicides, such as glyphosate [43]. This study utilized Attenuated Total Reflectance Fourier-Transform Infrared (ATR-FTIR) spectroscopy to investigate the structural changes in the metribuzin molecule, indicating its effective biodegradation by the actinobacterial isolates. The results underscore the capability of these microorganisms to modify the herbicide through the cleavage, formation, and rearrangement of critical functional groups. These insights enhance our understanding of the metabolic pathways and mechanisms involved in the microbial degradation of metribuzin, which is crucial for assessing the environmental fate and management of this commonly used agrochemical. While studies employing this analytical technique for the assessment of metribuzin biodegradation are limited, the present findings are consistent with the work of [54], who utilized FTIR to demonstrate the biodegradation of the pesticide atrazine by *Bacillus atrophaeus* YQJ-6.

## 5. Conclusions

The present study has identified four exceptional actinobacteria strains, namely, *Streptomyces toxytricini* strain CH, *Streptomyces stelliscabiei* strain B2, and *Streptomyces heliomycini* strains C1 and C3, isolated from agricultural soil in the Algerian Sahara. These strains have demonstrated remarkable capabilities in utilizing the herbicide metribuzin as their sole carbon source for growth, showcasing their potential as highly effective bioremediation agents. The optimization of key environmental conditions, such as pH 7.2, temperature at 30 °C, and an inoculum volume of 4%, has further enhanced the biodegradation performance of these actinobacteria isolates. The substantial reductions in total organic carbon (TOC) levels, reaching up to 75.99% for the C3 strain, provide compelling evidence of the complete mineralization of the herbicide by these microorganisms. The application of complementary analytical techniques, including ATR-FTIR analysis, has conclusively confirmed the structural modifications undergone by the metribuzin molecule during the biodegradation process. This comprehensive characterization, coupled with the quantitative kinetic studies, solidifies the conclusion that the *Streptomyces* strains isolated in this study possess exceptional abilities to degrade and mineralize the herbicide metribuzin. The findings of this study contribute significantly to the growing body of knowledge on the potential of actinobacteria as highly efficient bioremediation agents for the treatment of environmental contaminants, such as the widely used herbicide metribuzin. These remarkable microorganisms hold great promise for future applications in the remediation of sites impacted by this plant protection product, thereby promoting sustainable agricultural practices and environmental protection.

## Figures and Tables

**Table 1:** Isolates from El-Oued soil are able to grow using the herbicide metribuzin as the sole carbon source.

Strains	Metribuzin Concentrations (mg/L)					
	1	10	25	50	100	200
CH	+++	++	+++	+++	+	+
C1	+++	+++	+++	+++	+	+
B1	−	−	−	−	−	−
B2	+++	++	+++	++	+	+
C2	+++	+++	++	−	−	−
C3	+++	++	++	+++	−	−
C4	+++	+	+++	−	−	−
C	++	++	+	−	−	−
CB	+++	++	++	−	−	−
3G	+++	+	+	−	−	−
GH	−	−	−	−	−	−
GL	−	−	−	−	−	−

(+++) strong growth, (++) medium growth, (+) weak growth, (−) no/minimal growth.

**Table 2:** Physiological properties of actinobacterial strains (CH, B2, C1, and C3).

	**CH**	**B2**	**C1**	**C3**
Temperature				
25 °C	+	+	+	+
30 °C	++	++	++	++
37 °C	+	+	+	+
40 °C	−/+	−/+	−/+	−/+
pH				
2	+	+	+	+
5	+	+	+	+
7	++	++	++	++
9	+	+	+	+
12	+	+	+	+
NaCl				
2%	++	++	++	++
5%	+	−	−	−
9%	−	−	−	−
15%	−	−	−	−
Enzymatic activity				
Starch hydrolysis	+	+	+	+
Casein hydrolysis	+	−	+	−
Gelatin Hydrolysis	−	+	+	+
Catalase	+	+	+	+
Carbon source				
D-Fructose	−	+	+	+
D-Glucose	+	+	+	+
D-Galactose	+	+	+	+
Nitrogen source				
Proline	−	−	+	+
Arginine	−	+	+	+
Threonine	−	−	−	+
Histidine	+	−	+	−
Asparagine	−	+	+	+
Tyrosine	+	−	+	−
Methionine	+	+	−	+

(++)> strong growth; (+) weak growth. (−/+) minimal growth.

**Table 3:** Molecular identification of the actinobacterial strains (CH, B2, C1, C3).

Bacteria	Close Strain	Accession. N
CH	*Streptomyces toxytricini*	PP413749
B2	*Streptomyces stelliscabiei*	PP413746
C1	*Streptomyces heliomycini*	PP413747
C3	*Streptomyces heliomycini*	PP413748

**Table 4:** Second-order rate constant (k) and half-life (t_1/2_) for degradation of metribuzin (50 mg/L) by the strains CH, B2, C1 and C3.

Strains	k	t_1/2_	R_2_
CH	0.0054	3.7	0.96
B2	0.0044	4.54	0.83
C1	0.0066	3.03	0.99
C3	0.0051	3.92	0.87

## Data Availability

The data of this study are available upon reasonable request to the corresponding authors.

## Supplementary Materials

The following supporting information can be downloaded at: https://www.mdpi.com/article/10.3390/toxics12100709/s1, Figure S1. Reduction of total organic carbon TOC by actinobacteria CH, B2, C1, C3 during degradation of 50 mg/L metribuzin over 15 days of incubation.; Table S1: Chemical composition of culture media.; Table S2. Cultural characteristics of actinobacterial strains CH, B2, C1, and C3.
