# Supplementary material for: Soil Actinobacteria Exhibit Metabolic Capabilities for Degrading the Toxic and Persistent Herbicide Metribuzin
Source: Toxics. 2024 Sep 29;12(10):709. doi: 10.3390/toxics12100709 (PMC11511370; doi:10.3390/toxics12100709)
Supplement: Supplementary file 1 [file toxics-12-00709-s001.zip › toxics-3191257-supplementary.pdf]

toxics

MDPI

Table S1. Chemical composition of culture media.

**Bennett medium**	
**Glucose**	10 g
Yeast extract	1 g
NZ amine (casein digest)	2 g
Beef extract	1 g
Agar	20 g
Distilled water	1000 mL
pH	7.3
**Yeast extact malt Extract agar medium**	
Yeast extract	3 g
Malt extract	3 g
Glucose	10 g
Peptone	5 g
Agar	20 g
Distilled water	1000 mL
pH	7.2
**ISP2 medium**	
Yeast extract	4 g
Malt extract	10 g
D-glucose	4 g
Agar	20 g
Distilled water	1000 mL
pH	7.3
**ISP7 medium**	
Glycerol	15 g
L-asparagine	1 g
K_2HPO_4	0,5 g
NaCl	0,5 g
$FeSO_4.7H_2O$	0,01 g
Agar	20 g
Distilled water	1000 mL
pH	7.2
**ISP9 medium**	
(NH4)2SO4	2.64 g
KH2PO4	2.38 g
K2HPO4 3H2O	5.65 g
MgSO4 2H2O	1 g
Saline solution	1 mL
Agar	20 g
Distilled water	1000 mL
pH	7
**MSM medium**	
KNO_3	13.76 g/L
KH_2PO_4	1.78 g/L
Na_2HPO_4. $2H_2O$	4.66 g/L
Na_2SO_4	9.68 g/L
EDTA	10 mg/L
$FeSO_4.7H_2O$	5 mg/L

$MnCl_2.4H_2O$	1.22 mg/L
$ZnSO_4.7H_2O$	0.25 mg/L
$CuSO_4$. 5H2O	0.2 mg/L
$CaCl_2.2H_2O$	1 mg/L
$Na_2MoO_4.H_2O$	0.2 mg/L
eau distillée	1000 mL
pH	7.3

Table S2. Cultural characteristics of actinobacterial strains CH, B2, C1, and C3.

		**CH**	**B2**	**C1**	**C3**
**ISP2**	Growth	+++	+++	+++	+++
	Colony color	Pink	White	White	White
	Aerial mycelium	Pink	Dark grey	Dark grey	Dark grey
	Substrate mycelium	Light pink	Brown	Brown	Brown
	Pigmentation	Absent	Dark brown	Dark brown	Dark brown
**ISP7**	Growth	+	+	+	+
	Colony color	Pink	White	White	White
	Aerial mycelium	Pink	Grey	Grey	Grey
	Substrate mycelium	Absent	Brown	Brown	Brown
	Pigmentation	Absent	Dark brown	Dark brown	Dark brown
**ISP9**	Growth	+	+	+	+
	Colony color	Pink	White	White	White
	Aerial mycelium	White to pink	Light grey	Light grey	White to brown
	Substrate mycelium	Light pink	Brown	Brown	Brown
	Pigmentation	Absent	Dark brown	Dark brown	Dark brown
**Bennett**	Growth	+++	+++	+++	+++
	Colony color	Pink	White	White	White
	Aerial mycelium	Pink	Dark grey	Dark grey	Dark grey
	Substrate mycelium	Light pink	Brown	Brown	Brown
	Pigmentation	Absent	Dark brown	Dark brown	Dark brown
**Yeast extact malt Extract agar**	Growth	+++	+++	+++	+++
	Colony color	Pink	White	White	White
	Aerial mycelium	Pink	Dark grey	Dark grey	Dark grey
	Substrate mycelium	Light pink	Brown	Brown	Brown
	Pigmentation	Absent	Dark brown	Dark brown	Dark brown

(+++) strong growth; (+) weak growth.

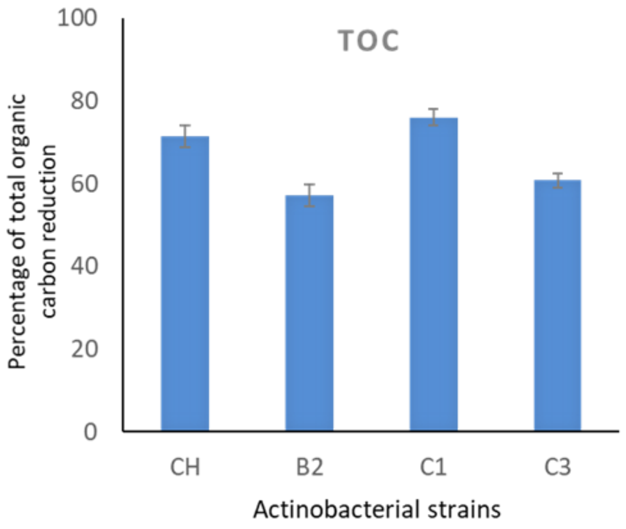

Figure S1. Reduction of total organic carbon TOC by actinobacteria CH, B2, C1, C3 during degradation of 50 mg/L metribuzin over 15 days of incubation.
